# IL-33/IL-31 Axis in Immune-Mediated and Allergic Diseases

**DOI:** 10.3390/ijms20235856

**Published:** 2019-11-22

**Authors:** Giuseppe Murdaca, Monica Greco, Alessandro Tonacci, Simone Negrini, Matteo Borro, Francesco Puppo, Sebastiano Gangemi

**Affiliations:** 1 Clinical Immunology Unit, Department of Internal Medicine, University of Genoa and Ospedale Policlinico San Martino, 16132 Genoa, Italynegrini@unige.it (S.N.); borromatteo@libero.it (M.B.); puppof@unige.it (F.P.); 2 Clinical Physiology Institute, National Research Council of Italy (IFC-CNR), 56124 Pisa, Italy; atonacci@ifc.cnr.it; 3 School and Operative Unit of Allergy and Clinical Immunology, Department of Clinical and Experimental Medicine, University of Messina, 98125 Messina, Italy; gangemis@unime.it

**Keywords:** allergy, cytokine, IL-33, IL-31, inflammation, autoimmune disease

## Abstract

Several allergic and immunologic diseases including asthma, food allergy (FA), chronic spontaneous urticaria (CSU), atopic dermatitis (AD), systemic lupus erythematosus (SLE), systemic sclerosis (SSc), rheumatoid arthritis (RA), and Behçet’s disease (BD) are characterized by the involvement of Th2 immunity. Several mediators lead to immunoglobulin (Ig)E production, thus including key cytokines such as interleukin (IL)-4, IL-5, and IL-13. Among them, IL-31 and IL-33 have been recently studied as novel biomarkers and future therapeutic targets for allergic and immunological disorders. IL-31 is a proinflammatory cytokine—it regulates cell proliferation and is involved in tissue remodeling. IL-33, acting through its receptor suppression of tumorigenity (ST2L), is an alarmin cytokine from the IL-1 family, whose expression is mediated by tissue damage. The latter has a pleiotropic effect, as it may modulate specific and innate immune cells functions. To date, several researchers have investigated the involvement of IL-31 and IL-33 in several allergic and immune-mediated diseases. Further studies are needed to understand the future applications of these molecules as novel therapeutic agents. This paper aims to give the readers a complete and updated review of IL-31 and IL-33 involvement among the most common autoimmune and allergic disorders.

## 1. Introduction

Allergic and autoimmune diseases are multifactorial conditions, in which both genetic and environmental factors play a crucial role. Although they are characterized by different phenotypes, these disorders often share a common and complex milieu of cytokines that are involved in their pathogenesis. Among them, interleukin (IL)-31 and IL-33 have been extensively studied. Indeed, since 2003, when IL-33 was firstly identified, many researchers investigated its functioning and its complex relations with other immune-regulatory pathways, suggesting that these inflammatory patterns were connected. Thus, this led to the newest theory of an “IL-31/IL-33 axis” that could be involved in several conditions such as allergies, autoimmune-diseases, and cancer [1,2,3,4,5].

## 2. From IL-33 and IL-31 “Single Molecules” to the Idea of an IL-31/IL-33 Axis

IL-33 is a member of the “alarmins” family. The family encompasses several endogenous peptides and proteins that are released in response to cellular damage, apoptosis, or immune activation.

Alarmins act as intercellular signals by interacting with chemotactic and pattern recognition receptors (PRRs) to boost immune cells in host defense. Moreover, on the basis of their ability to activate dendritic cells (DC) to mature ones, alarmins cooperate with adaptive immunity and T cell-dependent long term immune memory [6].

IL-33 is a tissue-derived nuclear cytokine produced by endothelial cells, epithelial cells, fibroblast-like cells, and myofibroblasts. It was recently hypothesized that IL-33 is a two-faced molecule. It can work both intracellularly as a nuclear factor able to regulate gene expression and extracellularly as an IL-1 family cytokine [1]. The ability to serve as an extracellular receptor able to activate immune cells is mainly due to its structure. Indeed, IL-33 is made up of two evolutionary conserved domains, the N-terminal nuclear domain and the C-terminal IL-1-like cytokine domain, divided by a divergent central part. Thanks to its IL-1 cytokine domain, IL-33 binds to its membrane receptor named ST2, which is a member of the toll-like/IL-1-receptor superfamily, to interact with IL-1 receptor accessory protein (IL1RAcP), a co-receptor made by a central five-stranded sheet rounded by five helices placed on the cytosolic end of the protein that is shared with other IL-1 family members (IL1α, IL1β, IL-36). The IL-33/ST2/IL1RAcP complex then induces the dimerization of the toll-interleukin receptor (TIR) domain, which leads to the activation of intracellular signaling through myeloid differentiation primary response 88 (MyD88) adaptor, interleukin receptor- associated kinase (IRAK)1 and IRAK4 kinases, and tumor necrosis factor receptor- associated factors (TRAF)6, which culminates in the activation of mitogen-activated protein (MAP) kinases and nuclear factor κB (NFκB) transcription factors, thus promoting the pro-inflammatory cascade. Moreover, this complex activates Jun kinase and extracellular signal-regulated kinase (ERK) expression, which downregulates forkhead box p3 (Foxp3) and GATA3 expression. As ST2 is mainly expressed by mast cells, group 2 innate lymphoid cells (ILC2s), eosinophils, and regulatory T cells (Tregs), these cells represent the major target of IL-33 [2]. Thus, this leads to the concept that IL-33 plays a crucial role in modulating immune cells functioning in several conditions such as asthma and lung diseases. The second molecule of interest is IL-31. This a gp130/IL-6 family cytokine with a four-helix bundle structure. IL-31 is mainly produced by cluster of differentiation (CD) 4+ T helper (Th2 cells), although mast cells and dendritic cells can produce it too but to a lower extent. The main targets of IL-31 are fibroblasts and eosinophils, which are activated through IL-31 receptor (IL-31R). To date, several isoforms of IL-31 receptor have been identified. Among them, CRL and IL-31 receptor alpha (RA)v2 are the soluble forms showing no transmembrane region, whereas IL-31RAv1 and IL-31RAv4 display the classical features of type I cytokine receptors, which are made of a cytokine receptor homology domain with two pairs of conserved cysteine residues and a WSDWS signature motif, followed by three fibronectin type III-like domains and a single transmembrane region connected to an intracellular tail. Within the cytoplasmic tail, there is a box-1 motif typically involved in the association with cytoplasmic tyrosine kinases of the Jak family [7,8]. The final signaling is then mediated by the binding of IL-31 receptor alpha (IL-31RA) and oncostatin-M receptor beta (OSMR), which are expressed on IL-31-activated monocytes.

IL-31 action is achieved through three signaling pathways: JAK/STAT pathway (Janus-activated kinase/signal transducer and activator of transcription), PI3K/AKT (phosphatidylinositol 3′-kinase/protein kinase) pathway, and MAPK (mitogen-activated protein kinase) pathway. Thus, the IL-31 receptor is mainly expressed in nonhematopoietic tissue, skin, and endothelium, suggesting that IL-31 has several functions in regulating these tissue responses. Indeed, several pieces of research have demonstrated that IL-31 stimulates pro-inflammatory cytokines, regulates cell proliferation, and is involved also in tissue remodeling [1,7,9,10].

Shortly after the discovery of IL-31 and IL-33, researchers investigated the possible relationship between these two molecules. Di Salvo et al. [1] published interesting research that highlighted the IL-33/IL-31 axis as a potential inflammatory pathway in allergic and inflammatory diseases. Researchers assumed that the presence of one interleukin might stimulate the induction of the other, amplifying inflammation and the consequent detrimental processes. Moreover, two experimental studies by Maier et al. [11] and Stott et al. [12] demonstrated the presence of a complex interplay between these two cytokines. Indeed, they noticed that IL-31 genetic expression and release from TH2 cells is induced by IL-4. Afterward, IL-33 enhances IL-4-induced IL-31 release. They also reported that IL-31 protein induction is mediated by IL-4/STAT6 and IL-33/NF-κB signaling and is downregulated by suppressor of cytokine signaling (SOCS)3.

To conclude, over recent years researchers’ attention has moved from the single molecule approach to a more complex idea of a structured pro-inflammatory axis. These discoveries help us, not only because they shed new light on disease pathogenesis, but also in that they improve more targeted therapies. In this review, we discuss some of the latest discoveries regarding IL-31 and IL-33 among allergic and autoimmune diseases.

## 3. Autoimmune Disorders

### 3.1. Behçet’s Disease

Behçet’s disease (BD) is a multi-systemic vasculitis with the highest prevalence among countries along the ancient Silk Road from the Mediterranean basin to East Asia.

The diagnosis is made on clinical criteria, and as to date there is no specific test, although an association with human leukocyte antigen (HLA)-B51 is a known genetic predisposition factor.

BD-typical features are recurrent oral and genital aphthous ulcers, ocular disease, and skin lesions. However, along with other systemic diseases, vascular, articular, gastrointestinal, neurologic, urogenital, pulmonary, and cardiac systems are often involved [13,14,15].

As is the case with other immune-related disorders, BD is characterized by multifactorial pathogenesis and several immunological abnormalities both regarding innate and humoral immunity have been detected. Among them, IL-1-related cytokines, thus including IL-33, have been recently studied. Indeed, Talei et al. [16] demonstrated that a specific polymorphism of the IL-33 gene, the rs1342326 T/G, may explain genetic susceptibility to BD, highlighting that this polymorphism may up-regulate IL-33 expression. Notably, Çerçi et al. [17] conducted a study to investigate the role of IL-33 among BD patients. They enrolled 54 BD patients, 31 had active BD and 23 had the inactive disease, and compared them with 18 healthy subjects to measure IL-33 levels using an enzyme-linked immunosorbent assay (ELISA). They found that serum IL-33 levels were significantly higher in patients with BD compared with the healthy controls (*p* < 0.01). Moreover, they noticed that among active BD patients with arthritis the mean serum IL-33 level was higher, but this finding was not statistically significant (*p* = 0.122). Another interesting study conducted by Kacem et al. [18] conducted on 40 BD patients demonstrated that messenger RNA (mRNA) expression of thymic stromal lymphopoïetin (TSLP) and IL-33 was increased in active BD with skin lesions. TSLP and IL-33 are both pro-inflammatory cytokines released from epithelial cells when facing stressing stimuli. Also, this represents the link between the environment and systemic immune responses.

High levels of IL-33 were also demonstrated in BD patients with neurologic involvement. Central nervous system (CNS) complications are rare but with high morbidity and mortality. Hamzaoui et al. [19] analyzed IL-33 levels in cerebrospinal fluid (CSF) of neuro BD (NBD), hypothesizing that this cytokine could be involved in oligodendrocyte and neuronal injury. They noticed that IL-33 levels were significantly higher in NBD patients compared to those who had the non-inflammatory neurological disease (NIND) and those with headache attributed to BD. Regarding the association between IL-31 and BD, data are lacking. However, as emerged from a study by Takeuchi et al. [20], IL-31 levels among BD patients with ocular involvement significantly reduced after infliximab (IFX) treatment. Thus, this suggests its role on disease course.

### 3.2. Systemic Lupus Erythematosus (SLE)

SLE is a multi-systemic disease characterized by the presence of several autoantibodies and immune dysregulations with a high prevalence in females [21,22]. Disease pathogenesis is still challenging as it is a multi-factorial condition in which several mechanisms are involved, including epigenetics [23]. Although great progress has been done on the development of new therapies, SLE patients still have great morbidity and mortality, which are mainly due to cardiovascular and renal involvement [24]. Among the plethora of immune-mediators that are currently under investigation, researchers recently focused on IL-33. Indeed, Yang et al. [25] conducted a study on 70 SLE patients, noticing that SLE patients had higher serum IL-33 levels compared to healthy controls. This study also highlighted that, although IL-33 may have a crucial role in the acute phase of the disease, specifically targeting erythrocytes and platelets, it was not associated with its course. Analogous results were obtained from a Guo et al. [26] study, as they noticed that IL-33 serum levels were higher in SLE patients. Moreover, they investigated the possible association between cytokine levels and clinical manifestations, noticing that there was a significant difference between IL-33 levels and C-reactive protein (CRP) levels and the erythrocyte sedimentation rate (ESR). Thus, this strengthened the idea that IL-33 may play a crucial role in the acute phase of the disease. Pre-clinical studies also hypothesized the role of IL-33 as an active player in SLE pathogenesis. Li et al. [27] conducted a study on lupus-prone mice, reporting that IL-33 inhibition may slow SLE through the expansion of T regulatory cells (T regs) and myeloid-derived suppressor cells (MDSCs) and inhibition of Th17 cells and proinflammatory responses. Thus, this indicated that the blockade of IL-33 has a protective effect on SLE. Genetic studies regarding IL-33 gene and its polymorphisms have also been conducted. Indeed, Zhu et al. [28] analyzed two IL-33 single nucleotide polymorphisms (SNPs), demonstrating that both were potential risk factors for developing SLE. On the other hand, at least two studies reported different results. Italiani et al. [29] conducted a study on IL-1 family molecules and SLE, and reported that IL-33 was significantly lower in SLE (*p* = 0.002), whereas soluble interleukin 1 receptor 4 (sIL-1R4), its natural inhibitor, was significantly higher (*p* < 0.0001). Moreover, they found no correlation between proteinuria and IL-33. Similarly, Mok et al. [30] analyzed 70 SLE patients’ sera, reporting that IL-33 level was not found to be related to lupus disease activity or specific organ involvement, nor sST2 level. On the other hand, serum sST2 level was significantly higher in active SLE patients compared with those who had an inactive disease (*p* = 0.02) and with normal controls (*p* < 0.001). To conclude, data regarding IL-33 and sST2 are still being debated, and perhaps further studies conducted on a wide number of IL-1-related molecules will shed more light on this topic.

### 3.3. Rheumatoid Arthritis (RA)

Rheumatoid arthritis (RA) is an autoimmune disease characterized by systemic inflammation of diarthrodial joints, which may lead to articular irreversible damage. It affects almost 1% of the global population and systemic involvement may be present. As is the case with other inflammatory diseases, several mediators are involved in RA pathogenesis, including IL-1 family members. Thanks to the discoveries of this field, new target therapies have entered the market with encouraging results [31,32,33].

Indeed, over recent years, evidence has been collected regarding a relationship between IL-33 and RA. Chen et al. [34], focusing on the protective role of IL-10, demonstrated that in mice IL-33 levels were down-regulated by IL-10. Therefore, they demonstrated that IL-33 expression, rather than its receptor (ST2) is positively correlated with IL-10 level in active RA. More recently, Macedo et al. [35] confirmed the triggering role of IL-33 in collagen-induced arthritis in experimental models. They demonstrated that the administration of interleukin-33 intensifies the process. Moreover, they found a correlation between cytokine concentrations in serum and synovial fluid of patients with RA and disease activity. Other interesting studies have been conducted to determine if there was any association between IL-33 levels and RA therapy.

Firstly, Sellam et al. [36] investigated IL-33 and rituximab (RTX), a genetically engineered chimeric mouse/human monoclonal antibody representing a glycosylated immunoglobulin with human IgG1 constant regions and murine light-chain and heavy-chain variable region sequences, which binds specifically to the transmembrane antigen, CD20, a non-glycosylated phosphoprotein, located on pre-B and mature B lymphocytes. The authors found that serum IL-33 may predict clinical response to RTX independently of auto-antibodies. Therefore, they proposed IL-33 as a new biomarker in addition to auto-antibody status in predicting RTX response in RA patients. An interesting study published by Choi et al. [37] evaluated the effects of tocilizumab IL-33 in patients with RA. Tocilizumab is a humanized monoclonal antibody that acts as an IL-6 receptor antagonist, which can be administered both intravenously or subcutaneously [38]. This study was conducted on 83 RA patients, and serum cytokine levels were analyzed at baseline and after 24 weeks of tocilizumab therapy. Data confirmed that IL-33 levels were significantly higher in RA patients than in healthy controls (*p* < 0.001). Moreover, a significant correlation with rheumatoid factor titer and IL-33 was found. Aside from this, the authors demonstrated that serum IL-33 levels decreased significantly after 24 weeks of tocilizumab therapy (*p* < 0.001), thus strengthening the concept that IL-33 could be used as a marker to monitor therapy response in RA.

On the other hand, a study by Rivière et al. [39] found that there was no association between IL-33 and response to tumor necrosis factor-alpha inhibitors (TNFi), as well as to non-TNFi drugs overall or analyzed separately (Table 2). Likewise, there was no difference when comparing the levels of serum IL-33 between responders and non-responders in TNFi and non-TNFi groups. To sum up, this study corroborates the association between serum IL-33 detection and seropositivity in RA patients. However, it did not reproduce the results obtained from the study by Sellam et al. [36].

### 3.4. Systemic Sclerosis (SSc)

Systemic sclerosis (SSc) is a complex disease characterized by fibrosis, vasculopathy, and immune dysregulation. Several systems may be involved, and disease triggers and pathogenesis are still under investigation. Indeed, actual therapies are organ-specific, but no curative therapies have emerged. New promising results came from autologous hematopoietic stem cell transplantation (AHSCT), however, it still stands as a major procedure with several complications, including infections, which leads to the concept that AHSCT could be proposed only to a small number of selected SSc patients [40,41,42]. As fibrosis is one of the cardinal characteristics of SSc, researchers have investigated its pathogenesis, noticing that IL-1 family cytokines are actively involved. Indeed, IL-33 may be considered as a biomarker of fibrosis involvement [43,44,45]. Zhang et al. [46] conducted a study on 56 Chinese SSc patients, reporting that IL-33 levels in SSc patients were significantly higher than in healthy controls. However, any significant correlation was found between cytokine levels and disease characteristics. On the other hand, Wagner et al. [47] found a significant correlation between IL-33 sST2 levels and skin involvement. They demonstrated that sST2 is elevated in late phase limited cutaneous SSc (lcSSc) as compared to patients with shorter disease duration or with the diffuse subtype of SSc. Moreover, they noticed that sST2 levels were decreased by prostanoid treatment. Analogous results were referred by Yanaba et al. [48]. Moreover, they reported that SSc patients with pulmonary fibrosis and decreased forced vital capacity presented higher IL-33 levels. With the aim of evaluating the relevance of IL-33 among interstitial lung diseases, Lee et al. [49] conducted a study among idiopathic pulmonary fibrosis (IPF) and other interstitial lung diseases, including non-specific interstitial pneumonia (NSIP), hypersensitivity pneumonitis (HP), and sarcoidosis, concluding that IL-33 levels detected in bronchoalveolar lavage fluids may be useful in differentiating IPF from other chronic interstitial lung diseases (ILDs). As the pro-fibrotic effect of IL-33 was well described, Koca et al. [50] analyzed IL-33 gene polymorphisms among the Turkish population to find a relationship with SSc. They found that rs7044343, a specific SNP, was higher in the SSc group compared to the control group, suggesting that the IL-33 gene may be a candidate gene to research in SSc.

**Figure 1:**
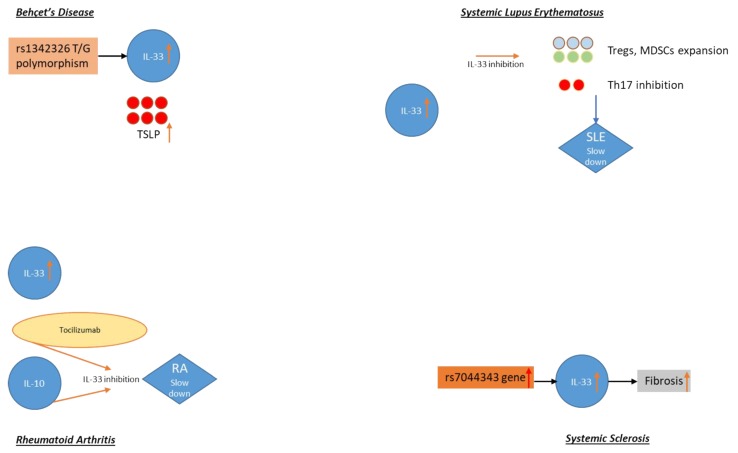
Interleukin (IL)-31 and IL-33 involvement in autoimmune disorders: Behçet’s disease (BD), systemic lupus erythematosus (SLE), rheumatoid arthritis (RA), systemic sclerosis (SSc). TSLP: thymic stromal lymphopoïetin, Tregs: regulatory T cells, MDSCs: myeloid-derived suppressor cells.

A brief, simple graphical overview of the IL-31 and IL-33 involvement in the autoimmune disorders above is shown in Figure 1, allowing the reader to understand at a glance their role in such conditions.

## 4. Allergic Disorders

### 4.1. Atopic Dermatitis (AD)

Atopic dermatitis (AD) is an inflammatory skin disease characterized by chronic or relapsing pruritus that may be accompanied by other atopic conditions such as asthma, food allergy, and rhino-conjunctivitis. AD prevalence is higher in children, but it can affect adults too [51]. AD pathophysiology is made of a complex net, in which both genetic and environmental factors play a role. One of the most important markers of AD is skin dehydration, which is caused by filaggrin mutations that mediate trans-epidermal water loss and pH alterations. Immune system dysregulation also takes part in AD development, thus leading to IgE-mediated hypersensitivity, contributing to skin disease pathogenesis [52]. Pro-inflammatory cytokines play a crucial role too. To date, there is no univocal explanation of IL-33 role among AD patients. Imai et al. [53] set up an animal model to investigate IL-33 expression and AD development. In their experiment, they demonstrated that transgenic mouse expressing IL-33 spontaneously develops AD with the activation of group 2 innate lymphoid cells (ILC2s) and basophils. Moreover, IL-33 proved to be a crucial modulator of eosinophil function [54]. More recently, Yi et al. [55] hypothesized the involvement of another molecule named intelectin (ITLN), which was found to be overexpressed both in asthmatic airways and in lesioned skin of AD, thus leading to the fact that ITLN contributes to allergen-induced IL-33 in asthma and AD [55]. On the basis of these studies, Peng et al. [56] evaluated the potential on the inhibition of atopic dermatitis (AD) of anti-mouse IL-33 antibody (alphaIL-33Ab) using a chemical-induced AD mouse model. They administered alphaIL-33Ab via subcutaneous injection to AD mice, whereas the control group received tacrolimus. AD-like mice treated with alphaIL-33Ab showed improved AD-like symptoms. Consequently, eosinophils and mast cell infiltration and serum IgE levels were also significantly reduced by alphaIL-33Ab, thus suggesting that blockade of IL-33 negatively influences AD expression. Results obtained from animal models were all confirmed in human cell line studies [57,58,59]. To sum up, it seems that a dysregulation of innate and adaptive immune response could lead to skin damage, which could induce an increased Th2 response (with an upregulated IL-31 and IL-33 production), thus leading to the AD worsening (i.e., scratching, wounding, infections) and progression. Among IL-1 family molecules, IL-31 has also been studied, and new therapeutic strategies have already entered the market or are near to doing so. It is known that IL-31 is one of the major promoters of pruritus and scratching behavior among AD patients. As suggested by Singh et al. [60], IL-31 activation may induce epidermal cell proliferation and thickening, which can lead to impaired skin barrier function in the pathological remodeling of the skin. Stating that IL-33 induces IL-31 expression, it is easy to understand how relevant this cytokine link is to AD development and maintenance. Both these molecules explain the itch–scratch cycle of AD. The first pharmacological studies focused on IL-4 inhibition. IL-4 induces the gene expression and release of IL-31 from human TH2 cells, and IL-33 further potentiates the IL-4-induced IL-31 release [9]. On this basis, dupilumab, a human monoclonal antibody against interleukin-4 receptor alpha, entered the market with great success [61]. New efforts are now focusing on nemolizumab (CIM331), a humanized antibody against interleukin-31 receptor A, with great expectations on targeted-therapy of AD [62]. To conclude, current theories concerning IL-31 expression among AD have highlighted the role of Th2 cells as one of the main producers of IL-31.

### 4.2. Allergic Contact Dermatitis (ACD)

Allergic contact dermatitis (ACD) is a skin disease in which T cell-mediated immune response is directed against the subject’s skin when exposed to allergens. Often, ACD can be accompanied by irritant contact dermatitis. Symptoms may vary from benign and self-resolving forms to diffuse skin damage, thus causing medical and socioeconomic problems. Several treatments have been developed to treat ACD, such as topical steroids, calcineurin inhibitors, phototherapy, retinoids, and immunosuppressive agents, but to date targeted therapies are still lacking [63]. Several studies have demonstrated that both IL-31 and IL-31 are involved in ACD pathogenesis [64]. Indeed, experimental mice models demonstrated that IL-33 blockade worsened contact hypersensitivity, and, on the other hand, injection of IL-33 inhibited contact hypersensitivity and induced Treg [65]. Aside from this, a study by Wang et al. [66] confirmed these results, noticing that IL-33 plays an anti-inflammatory effect targeting miR-155 in mast cells. Moreover, a study conducted in a mouse model of poison ivy ACD showed that IL-33/ST2 signaling is present in primary sensory neurons and promotes pruritus in affected mice [67]. The involvement of IL-31 and IL-33 was also confirmed among human models. More specifically, IL-31 was found to be expressed in skin biopsies of ACD patients, whereas IL-33 was induced in keratinocytes. Guarneri et al. [64] analyzed serum levels of both cytokines among ACD patients, finding that IL-31 levels were significantly higher in patients than in controls. On the other hand, IL-33 serum levels were not different between patients and controls. Moreover, in their study, they confirmed that IL-31 expression was related to pruritic symptoms, whereas IL-33 stands as an early warning system of skin damage.

### 4.3. Asthma and Allergic Rhinitis

Asthma is a chronic inflammatory respiratory disease characterized by reversible airflow obstruction on spirometry. Although several treatments are now available, asthma exacerbations still represent a cause of morbidity and mortality with a great impact on social and economic aspects [68]. It can affect both children and adults, and its pathogenesis relies on multiple factors. More specifically, asthma may be distinguished between atopic and non-atopic, depending on its trigger. As a complex disease, both Th1 and Th2 inflammatory pathways are involved, thus explaining the great heterogenicity of detectable cytokines. Several studies have demonstrated that among the aforementioned molecules, IL-33 has a crucial role, as it was shown to be a mediator of inflammation and fibrotic damage [69]. Moreover, some researchers have noticed that specific IL-33 gene polymorphisms may explain the different disease phenotype [70]. As highlighted by Bhowmik et al. [71], IL-33 can be a useful biomarker to detect atopic asthma. Indeed, researchers found that IL-33 was significantly up-regulated (3.84-fold) in atopic asthmatic patients compared to healthy controls. The same results were obtained by Jackson et al. [72]. On the basis of the concept that rhinovirus is one of the most common asthma exacerbation triggers, they built up a human experimental model in order to detect IL-33 levels during the rhinovirus infection in asthmatic and healthy airways. They noticed that IL-33 was significantly increased by rhinovirus infection, thus suggesting that IL-33 inhibition could be a new successful therapy for asthma. More recently, Allinne et al. [73] developed an experimental murine model of severe airway inflammation. Researchers administered an IL-33 neutralizing antibody, showing that both airway remodeling and inflammation improved, thus concluding that IL-33 blockade may be a new target to focus upon in order to decrease asthmatic exacerbations. Recently, the relationship between asthma and IL-31 was also studied. Ip et al. [74] demonstrated that this cytokine is greatly involved in bronchial inflammation, acting through a complex net of cellular mediators. Indeed, Edukulla et al. [75], using a model of in vivo allergic asthma induced by soluble egg antigen, noticed that the absence of type II IL-4 receptor signaling is sufficient to attenuate the expression of IL-31RA, thus suggesting that Th2 cytokines are the main triggers of IL-31RA expression and play a crucial role in Th2-mediated IL-31/IL-31RA connections. Analogous results were obtained by Huang et al. [76], who studied IL-31 and IL-31RA levels during eight consecutive ovalbumin (OVA) challenges, confirming that the cytokine levels were consistently high after a period of weeks. Concordant data were also obtained from human studies. In fact, Lai et al. [77] compared IL-31 levels in the serum, bronchoalveolar lavage fluid (BALF), and bronchial tissue specimens of asthmatics to healthy subjects. They demonstrated that serum and BALF IL-31 levels were significantly elevated in patients with asthma compared with controls. Moreover, cytokine levels were directly proportional to disease severity. Data concerning the high serum levels of IL-33 and IL-31 levels were also gained from patients affected by the combination of allergic asthma and rhinitis [78]. More specifically, Moaaz et al. [79] conducted a study on 110 Egyptian asthmatic children compared to 50 healthy controls, noticing that IL-31 levels were higher in the first group. Moreover, analyzing the sub-classes of asthmatic patients, they found that IL-31 was higher in the atopic asthma group. A study by Vocca et al. [80] was also in line with these results. In fact, they found significant correlations between plasmatic components of the IL-33/ST2 axis and IL-31 in both allergic rhinitis patients and those with concomitant allergic asthma. Analogous results were also observed in a pediatric population [81]. To sum up, both Th1 and Th2 responses are linked to IL-31 and IL-33 expression among asthmatic patients, and, more specifically, the pathogenetic role of IL-33 can be explained with the activation of Th2 cells, which leads to this cytokine increasing with a subsequent inflammatory effect on bronchi.

### 4.4. Chronic Spontaneous Urticaria (CSU)

Chronic spontaneous urticaria (CSU) is a common skin disorder characterized by the appearance of pruritic wheals lasting <24 h for a time period of at least 6 weeks and often for decades. Depending on its duration it can be classified as acute or chronic. Although CSU has a favorable prognosis, it represents a highly invalidating disease, as pruritus, which is its main symptom, may induce sleep disorders, thus reducing quality of life and work performance. CSU pathogenesis is multi-factorial and an underling immune-disease can also trigger CSU [82].

Both IL-31 and IL-33 were shown to be involved in CSU pathogenesis [83]. Notably, studies conducted on skin biopsies of CSU patients demonstrated significantly higher levels of IL-33 compared to healthy controls, thus suggesting that the expression of Th2-promoting cytokines may have a crucial role in whealing [84]. On the other hand, a study conducted by Puxeddu et al. [85] on IL-33/sST2 levels showed that there was no difference between cytokine levels among CSU patients compared to healthy controls. Several studies confirmed the role of IL-31 as a mediator of pruritic symptoms [10]. Indeed, until recent years, histamine and neuropeptides were considered the main causative agents of pruritus. However, novel studies have demonstrated that Th-2 cells play a crucial role too, thus including basophils, which were demonstrated to be a source of IL-31. Notably, in their study, Raap et al. [86] analyzed IL-31 expression in skin samples derived from CSU patients, noticing that IL-31 was highly expressed in the skin of CSU patients and was released from isolated basophils following either anti-IgE, IL-3, or *N*-formylmethionyl-leucyl-phenylalanine (fMLP) stimulation. Besides skin specimens, high levels of IL-31 were also found on CSU patients serum samples [87,88].

To sum up, both IL-31 and IL-33 are involved in CSU pathogenesis and expression. However, the role of IL-33 is still unclear, whereas IL-31 is already considered a well-defined pharmacological target [89].

### 4.5. Food Allergy (FA)

Food allergy is a common disease, whose prevalence has increased during recent decades, affecting both adults and children. The clinical characteristics of FA are heterogeneous and often unpredictable, as they depend on a wide range of different factors. Indeed, FA symptoms may range from oral allergic syndrome to life-threating anaphylaxis. Therefore, FA diagnosis and correct management are mandatory. Several studies have investigated FA pathogenesis, and recently researchers have shed new light on the involvement of cytokines and small molecules [90]. Among them, the role of IL-33 has been extensively studied. Indeed, some studies demonstrated that IL-33 expression is increased after antigen stimulation, thus promoting Th2 responses [91,92]. A recent study by Khodoun et al. [93] conducted on FA-induced mice demonstrated that IL-25, IL-33, and TSLP inhibition through specific monoclonal antibodies (mAbs) strongly inhibited FA development. More specifically, researchers noticed that single mAbs were unable to induce FA, whereas the combination of three achieved the objective. On the other hand, single mAbs were capable of maintaining FA. Thus, authors hypothesized that combined treatment with antagonists to all three pro-TH2 cytokines or with an inhibitor of pro-TH2 cytokine production might be able to suppress established human FA. Analogous results were obtained from a study by Han et al. [94]. They noticed that, when stimulated with specific antigenic stimuli, mice lacking IL-33 signaling did not develop atopic symptoms, neither cutaneous nor gastrointestinal. Moreover, they noticed that those mice who presented gastrointestinal symptoms recovered after IL-33 blocking. The role of IL-33 was also studied in murine models presenting with FA-induced anaphylaxis. Indeed, Galand et al. [95] evaluated wild-type mice that were epicutaneously sensitized with ovalbumin (OVA) and then challenged orally with OVA. They found that IL-33 promotes oral anaphylaxis after epicutaneous sensitization by targeting MCs. Therefore, therapy targeted to IL-33 blockade might prevent food-induced anaphylaxis in atopic patients.

**Figure 2:**
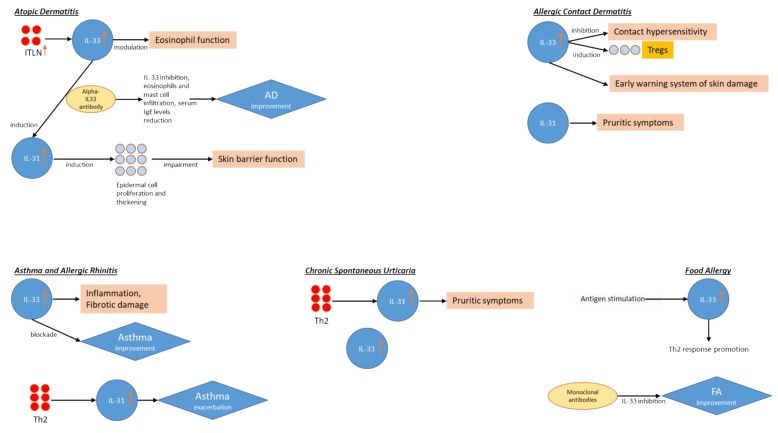
IL-31 and IL-33 involvement in allergic disorders: atopic dermatitis (AD), allergic contact dermatitis (ACD), asthma and allergic rhinitis, chronic spontaneous urticarial (CSU), and food allergy (FA).

A brief, simple graphical overview of the IL-31 and IL-33 involvement in the aforementioned allergic disorders is shown in Figure 2, which eases the reader in understanding at a glance the role of IL-31 and IL-33 in such conditions.

## 5. Conclusions

To summarize, our review shows that the IL-31/IL-33 axis represents a potential pathway of inflammation in allergic and autoimmune diseases. In particular, the activation of the IL-33/ST2-involving Th2/IL-31 immune response has a crucial role for the development of allergic inflammation, such as in asthma. In future, the dosage of these cytokines could be useful for the diagnosis, staging, and monitoring of the therapeutic efficacy in various allergic and autoimmune diseases.

In the light of these considerations, the pharmacological control of IL-33/ST2 activity may be crucial in the development of novel therapeutic approaches for the treatment of these inflammatory diseases.

To sum up, the aim of this paper review is to give the readers a new and updated review of this topic, focusing on the major immunologic and allergic disorders.
